# Influence of families and other adult support on HIV prevention outcomes among black men who have sex with men

**DOI:** 10.1186/s12889-024-18171-z

**Published:** 2024-03-15

**Authors:** Donte T. Boyd, S. Raquel Ramos, Allysha C. Maragh-Bass, Typhanye V. Dyer, Edem Yaw Zigah, Gamji Rabiu Abu-Ba’are

**Affiliations:** 1 College of Social Work, The Ohio State University, 1047 College RD, #325K, Columbus, OH 43215 USA; 2 Center for Interdisciplinary Research on AIDS (CIRA), Yale University School of Public Health, New Haven, CT USA; 3 University of California Center for AIDS Prevention Studies, University of California, San Francisco, San Francisco, CA USA; 4 School of Nursing, Yale University, Orange, CT USA; 5 FHI 360, Durham, NC USA; 6 Duke Global Health Institute, Duke University, Durham, NC USA; 7 Department of Epidemiology and Biostatistics, School of Public Health, University of Maryland, College Park, MD USA; 8 Behavioral, Sexual, and Global Health Lab, Jama’a Action, West Legon, Accra, Ghana; 9 School of Nursing, University of Rochester, Rochester, NY USA

**Keywords:** PrEP use, BMSM, Families, Condom use, HIV Testing

## Abstract

**Background:**

Prior research has consistently shown that the involvement of families plays a vital role in reducing risk behaviors, such as engaging in condomless sex, and promoting HIV prevention behaviors among young Black men who have sex with men (YBMSM). With the aim of expanding the existing knowledge, this study aimed to examine the specific influence of families and other supportive adults in facilitating casual condom use, partner condom use, HIV testing, and preexposure prophylaxis (PrEP) utilization among young Black MSM.

**Methods:**

A sample of YBMSM aged 18–29 years (*N* = 400) was collected online. We used a path analysis to examine the influence of family factors on PrEP stigma and PrEP use. Respondents were recruited from December 1, 2021, to January 31, 2022. We used a path analysis to examine the direct and indirect effects of family factors on PrEP use through HIV testing and encouraging condom use.

**Results:**

Among BMSM, other adult support was positive and directly associated with condom use by both casual partners (β = 0.04, *p* < .05) and partners (β = 0.17, *p* < .01). Condom use by casual partners was negative and was directly associated with HIV testing (β = − 0.15, *p* < .01).

**Conclusion:**

The primary aim of this research was to examine the influence of family and adult support on HIV prevention behaviors among young Black MSM, including condom use, HIV testing, and PrEP use. Our findings highlight the significance of implementing interventions that incorporate families and other supportive adults to enhance the engagement of young Black MSM in HIV prevention behaviors.

## Background

In recent years, despite advances in HIV prevention, the incidence of new HIV infections among young Black gay men aged 18–29 years has remained disproportionately high compared to other racial and ethnic groups [1]. The impact of systemic racism on health care and social determinants of health, and ongoing marginalization of Black communities in the United States, has led to disparities in access to education, employment opportunities, housing, and health care, which contributes to a lack of awareness and resources for HIV prevention [2–4]. Furthermore, the intersectionality of race and sexual identity creates a unique set of challenges regarding HIV prevention among young Black gay men [5–7].

A biomedical solution that has gained increasing popularity is preexposure prophylaxis (PrEP), a daily medication that can be taken orally or via injection and can significantly reduce the risk of acquiring HIV. However, PrEP use among eligible young Black men who have sex with men (YBMSM) remains markedly low [8]. This is due to systemic structural barriers, such as limited access to health care, inequitable distribution of HIV prevention resources in the health care setting, as well as social and cultural factors that may influence PrEP perceptions, attitudes, and behaviors [9]. Additionally, the stigma associated with HIV and PrEP use, mistrust of the health care system, lack of clinicians with LGBTQ + competency, concerns about confidentiality and privacy, and discrimination based on race and sexual identity [6] have created a macrocosm of HIV health inequities in this population, which further increases HIV risk. The cyclical nature of these phenomena leaves YBMSM in a whirlwind of disparately poor health care outcomes.

Young Black MSM employ a plethora of HIV prevention strategies, including behavioral strategies (e.g., condom use) and biomedical strategies (e.g., PrEP use) [10]. Condoms are a means of preventing both sexually transmitted infections and HIV, and condom use has been widely accepted as an effective prevention strategy [11]. However, condom use continues to be a challenge despite widespread availability: only 25–28% of men who have sex with men (MSM) report using condoms consistently [10–13]. Factors contributing to low condom use include fear of HIV stigma and discrimination from various sources, as well as cultural and religious beliefs that view same-gender attractions as immoral or sinful [14–15]. These beliefs may lead to a reluctance to openly address sexual identity and behaviors with family members, as well as subsequent avoidance of seeking HIV prevention services, including education on condom and PrEP use. Ending the HIV epidemic requires an urgent push to employ all HIV prevention strategies. Previous research suggests that these strategies are not abandoned when MSM use PrEP but instead are adapted to accommodate the addition of another HIV prevention strategy, such as using strategies situationally rather than with every partner [11].

Effective sexual health communication through family bonding or other adult support is critical to addressing the unique challenges in HIV prevention faced by YBMSM and is an essential aspect of social support [16–17]. According to one study, sexual health communication reduced the perceptions of HIV stigma in a national sample of young adults [18]. Family support can provide an overall sense of well-being, belonging, and acceptance, which creates an environment of safety to discuss one’s feelings with their loved ones. However, many BMSM may delay or avoid disclosing their sexual identity to their family for fear of rejection, homophobia, or being ostracized by family members or their communities, which can result in poor mental health and increased HIV risk [18–19]. Young MSM experiencing these challenges may seek social support from other adults in their communities, or nonbiological families that assume the role of family members and provide love, acceptance, and social support, which has been an important cornerstone for sexual minority populations [20]. In addition, having outside adult support has been increasingly recognized as an important form of social support for YBMSM. However, further study is required to explore the role of other adults who support and value YBMSM and sexual health communication related to the prevention of HIV and sexually transmitted infections.

Studies have shown that family bonding and having positive adult support, in general, can act as a protective factor against HIV by promoting healthy communication regarding sex and substance use, as well as providing emotional support [17–21]. The purpose of this study was to investigate the role of families of origin and chosen families in encouraging casual condom use, partner use, HIV testing, and PrEP use among YBMSM aged 18–29 years, using the ecodevelopmental theory as a guide.

## Theoretical framework

### Ecodevelopmental theory

The ecodevelopmental theory is based on Bronfenbrenner’s social-ecological model, which frames an individual’s social ecology in the context of four interrelated systems: microsystem, mesosystem, exosystem, and macrosystem [21–25]. Unlike the social-ecological theory, ecodevelopmental theory emphasizes the role of family functioning and interactions on risk and protective processes from a developmental perspective. We apply the ecodevelopmental theory on both risk and protective processes to investigate how the family context (e.g., family bonding and open family community) influences condom use, HIV testing, and PrEP use, both directly and indirectly. To date, the ecodevelopmental theory has proved beneficial in defining the influence of family factors on HIV attitudes and HIV prevention outcomes [24–25]. In addition, we extend the ecodevelopmental theory to include chosen family members and their influence on HIV prevention outcomes for BMSM.

It is critical to understand how family factors influence HIV prevention behaviors, such as PrEP use among Black MSM. Having supportive families and adults has been found to be a protective mechanism against stigma, oppression, and improve health outcomes, including HIV prevention outcomes for Black youth and sexual minorities [16, 26]. Understanding BMSM as individuals within the family context can help increase awareness of HIV, potentially reduce HIV stigma, and increase HIV prevention uptake. The ecodevelopmental theory can enhance our understanding of oppression and disparities in public health in the family context and can highlight the importance of both chosen families and families of origin in the lives of BMSM.

### Current research

**Fig. 1:**
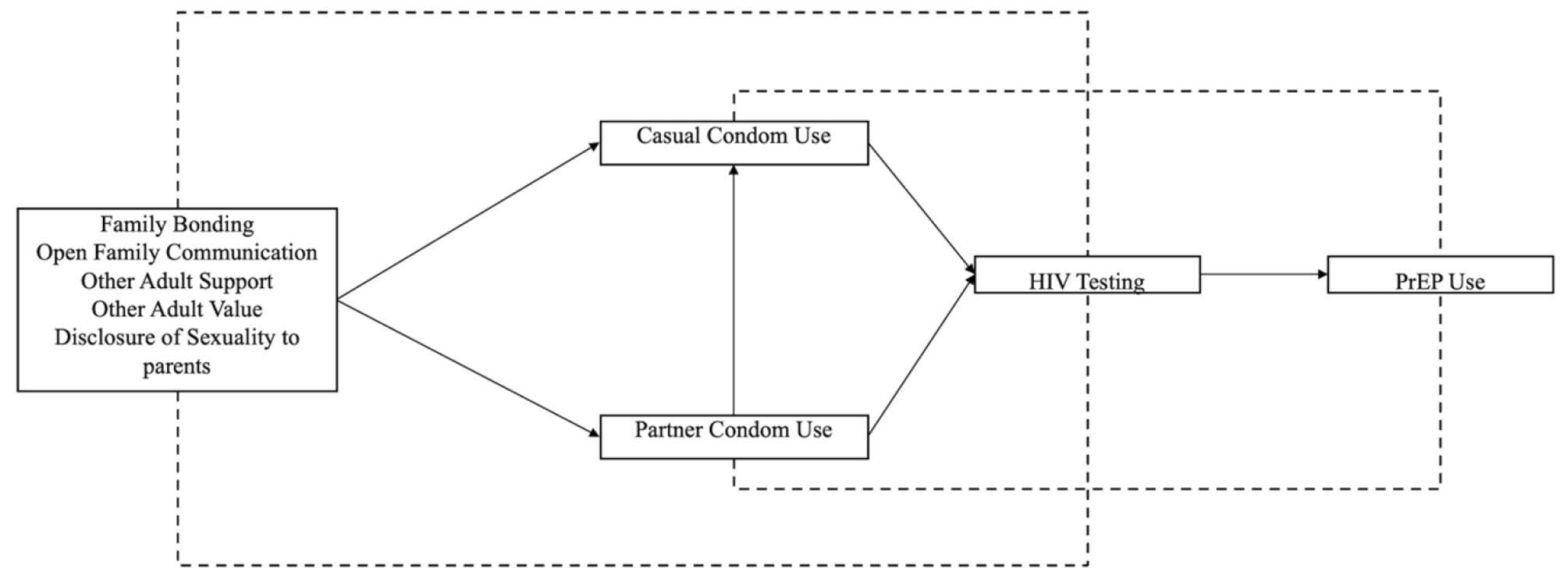
An illustration of the hypothesize model on how family factors influence, other adult support and value, and disclosure of sexuality to parents directly and indirectly influence casual condom use, partner condom use, HIV testing and PrEP use

Previous research has demonstrated that families and positive adult support for youth and young adults can contribute to reducing risk behaviors, such as condomless sex, and increasing HIV prevention behaviors, such as HIV testing [17, 21]. This is one of the first studies to use the ecodevelopmental theory to investigate the role of families of origin and chosen families in encouraging casual condom use, primary partner condom use, HIV testing, and PrEP use among YBMSM aged 18–29 years. Our first hypothesis was that family factors (family bonding, open family communication), other adult support, other adult value, and disclosure of sexuality to parents would be associated with an increase in casual and partner condom use. Our second hypothesis was that both casual and partner condom use would be associated with an increase in HIV testing. Our third hypothesis was that family factors, other adult support, other adult value, and disclosure of sexuality to parents would be indirectly associated with HIV testing and PrEP use. Lastly, our fourth hypothesis was that casual and partner condom use would be indirectly associated with PrEP use (see Fig. 1).

## Materials and methods

### Study procedures and recruitment

This study utilized data from a larger research project that aimed to explore strength-based strategies in promoting sexual, physical, and mental health among young Black MSM aged 18–29 years [17]. The survey was designed using Qualtrics software. Recruitment flyers containing an anonymous survey link were disseminated through social media platforms such as Facebook and Twitter, as well as community-based organizations and Amazon Mechanical Turk (MTurk) [27]. The primary investigator and research assistants consistently shared the survey link every morning at 8 a.m. Eastern Standard Time across social media channels.

MTurk was utilized as a cost-effective and efficient method for recruiting participants across various disciplines, including public health [28]. To be eligible for the survey, individuals had to be registered with MTurk, have a minimum approval rating of 95% based on past surveys, be 18 years or older, and reside in the United States, as confirmed during their initial MTurk registration [28–31]. Those who logged into the MTurk platform during the survey’s week were notified about an opportunity to participate in a survey focused on strength-based approaches to sexual, physical, and mental health among young Black men who have sex with men aged 18–29. Participants were informed that the survey would take approximately 20 min to complete and would be released every morning at 8 a.m. Eastern Standard Time. They were required to complete the survey in one session and were compensated $1 for their time, in addition to other incentives provided by MTurk [28–31].

For community-based organizations, the research team shared the flyer with community health workers, and they distributed the flyer to eligible participants who were considered clients of their organization. Participants were recruited from December 1, 2021, to January 31, 2022. All individuals who completed the 20-minute survey and provided an email address received a $35 electronic Amazon gift card.

To ensure the quality of the data and minimize the impact of bots, the survey utilized Qualtrics survey protection features. The research team implemented several measures to verify the respondents’ location in the United States and ensure data integrity. This included checking IP addresses, preventing the same respondent from answering eligibility or survey questions multiple times, and excluding participants who failed the speeding check (those who completed the survey significantly faster than the median duration). Qualtrics survey protection also incorporated tools such as ballot box stuffing prevention, reCAPTCHA scores (where respondents were asked to identify items in pictures or replicate a series of letters), and bot detection features. These measures helped detect and mitigate the influence of bots on the survey responses.

Participants were directed to the survey link where they were presented with an informed consent form and informed consent was obtained from all study participants. Prior to proceeding with the survey, participants were asked to complete a screening tool to determine their eligibility for the study. Those who met the inclusion criteria were then prompted to provide information on demographics, such as age, gender, and race/ethnicity. The survey covered various topics including strength-based assets such as family support and communication, as well as sexual, physical, and mental health. Those who accessed the survey through social media platforms or MTurk completed it using their personal computers. For participants who completed the survey at a community-based organization, computers or tablets provided by the organization were used. Ethical approval for the study was obtained from the Ohio State University Institutional Review Board (IRB # 2021E1175).

### Participants

The inclusion and exclusion criteria were consistent across all sampling sites. Eligible participants for the study were required to self-identify as Black or African American, be between the ages of 18 and 29, reside in the United States, have been assigned male at birth, fluent in English, currently identify as a male, and have engaged in sexual contact (oral, anal, or otherwise) with a male within the past year. Any respondents who did not meet these criteria were promptly exited from the survey. To ensure comprehensive data collection, the survey incorporated Qualtrics’ forced response option, which ensured that every participant had to answer each question.

The final sample included 400 YBMSM aged 18–29 years (M = 23.46; SD = 2.59). Most participants (*n* = 200) were recruited through MTurk, followed by community-based organizations (*n* = 100) and social media platforms (*n* = 100). The sample primarily identified as Black American or African American (75%), with smaller proportions identifying as Caribbean (10%) and Afro-Latino (10%), while 5% self-identified as continental African. In terms of educational attainment, 28% of the sample had not completed high school, while 29% had completed college or postgraduate studies. The average household income ranged from less than $20,000 to $150,000, with the average household income being $58,000.

### Measures

#### Outcome variable

PrEP use was measured using a single item based on a dichotomous response (0 = *No, I haven’t taken PrEP* and 1 = *Yes, I am currently taking PrEP*). Participants were asked the following question and given the following definition: “Have you ever taken PrEP (preexposure prophylaxis) in the past 12 months? PrEP (preexposure prophylaxis) is medicine people at risk for HIV take to prevent getting HIV from sex or injection drug use.”

#### Mediators

HIV testing, in this study, was based on the participants’ responses to the following: “Have you, yourself, ever been tested for HIV in the last 12 months?” The responses were coded as zero for a negative response and 1 for an affirmative response.

Primary partner condom use was measured using a single item ranging from zero (*no*) to 1 (*yes*). Participants were asked the following question: “During the last 12 months, did you top or bottom with your primary or main male partner with a condom (e.g., you put your penis in his anus or butt)?”

Casual partner condom use was measured using a single item ranging from zero (*no*) to 1 (*yes*). Participants were asked the following question: “During the last 3 months, did you top or bottom with a casual male sex partner with a condom (you put your penis in his anus or butt)?”

#### Independent variables

Family bonding was measured using three items based on a 5-point Likert scale ranging from 1 (*strongly disagree*) to 5 (*strongly agree*). Participants were given statements such as “My parents give me help and support when I need it.” We averaged responses to these three items, with higher scores indicating more family support [16, 21]. The Cronbach’s alpha was 0.95.

Open family communication was measured using a single item on a 5-point Likert scale-type question ranging from 1 (*strongly disagree*) to 5 (*strongly agree*). Participants were asked to respond to the following statement: “I have lots of good conversations with my parents” [32].

Communication about sex and drugs with parents was measured using a single item ranging from 1 (*never*) to 5 (*all of the time*), which asked respondents the following: “If you had an important concern about drugs, alcohol, sex, or some other serious issue, would you talk to your parent(s) about it?” [32].

Other adult support was measured by using a single-item, 5-point Likert-type question, with values ranging from 1 (*strongly disagree*) to 5 (*strongly agree*), asking participants to rate the statement “Adults in my town or city listen to what I have to say,” with a higher score indicating that adults in the community listen to the young men [32].

Other adult value was measured by using a single-item, 5-point Likert-type question, with values ranging from 1 (*strongly disagree*) to 5 (*strongly agree*), asking participants to rate the statement “Adults in my town or city make me feel important,” with a higher score indicating that adults in the community support the young men [32].

### Statistical analysis

Table 1 presents frequencies and percentages of categorical variables. Table 2 provides means and standard deviations for the study variables. We first conducted a correlation analysis to examine the relationships between the key study variables, as shown in Table 3. Next, we utilized M-Plus version 8.7 to conduct a path analysis, which allowed us to assess the direct and indirect effects of family factors, other adult support and value, disclosure of sexuality to parents on condom use, HIV testing, and PrEP use among young Black men who have sex with men (YBMSM) aged 18–29 years. The means and variance-adjusted weighted least squares estimator was used instead of maximum likelihood estimation, as this estimator is preferred when the dependent variable is categorical and the data are not normally distributed [33]. The percentage of missing data was less than 5%. Full information maximum likelihood was used for missing data [34]. The goodness-of-fit was assessed with measures using the chi-square test, Akaike information criterion (AIC), and Bayes information criterion (BIC) because the dependent variable was categorical. In addition, standardized beta coefficients and *p*-values were included and used to examine associations among study variables.

**Table 1:** Frequency and percentages of categorical study variables (*N* = 400)

Variable	Frequency	Percent
**Preexposure prophylaxis use**		
No	100	29
Yes	250	71
**HIV testing**		
No	100	29
Yes	250	71
**Casual condom use**		
No	150	43
Yes	200	57
**Partner condom use**		
Yes	175	50
No	175	50
**Disclosed sexuality to parents**		
Yes	200	57
No	150	43

**Table 2:** Means, standard deviations, and ranges of continuous variables (*N* = 400)

Variable	Mean	Standard deviation	Range
Age	23.0	2.59	18–29
Family bonding	3.78	1.01	1.0–5.0
Open family communication	3.84	1.07	1.0–5.0
Other adult support	3.77	1.00	1.0–5.0
Other adult value	3.72	0.89	1.0–5.0

**Table 3:** Bivariate correlations of study variables (*N* = 400)

Preexposure prophylaxis use	1							
HIV testing	0.14*	1						
Partner condom use	0.25***	0.03	1					
Casual partner condom use	0.20***	−0.11**	0.40***	1.				
Family bonding	0.06	−0.09	0.04	−0.05	1.			
Disclosed sexuality to parents	0.05	0.13**	−0.07	0.01	0.20***	1		
Other adult value	0.09	−0.09	−0.02	0.01	0.59***	0.01	1	
Other adult support	0.13**	−0.04	0.08	0.02	0.57***	0.13*	0.56***	1
Open family communication	0.12*	−0.16***	0.06	−0.03	0.79***	0.16**	0.54***	0.53***

Note: *p < .05, **p < .01, ***p < .001

## Results

A total of 81% of the sample reported using PrEP and 78% reported receiving an HIV test (Table 1). The mean age of the sample was 23 years (*SD* = 2.59). Results indicated that YBMSM reported higher levels of family bonding (*M* = 3.78, *SD* = 1.01), open family communication (*M* = 3.84, *SD* = 1.07), other adult support (*M* = 3.77, *SD* = 1.00), and other adult value (*M* = 3.72, *SD* = 0.89). Communication with parents about sex and drugs was moderate (*M* = 3.41, *SD* = 1.22) (Table 2).

Correlation results (see Table 3) indicated that HIV testing was positively correlated with PrEP use (*r* = .14, *p* < .05). Partner condom use was positively associated with PrEP use (*r* = .25, *p* < .001). Casual partner condom use was positively associated with PrEP use (*r* = .20, *p* < .001) and partner condom use (*r* = .40, *p* < .001), and negatively associated with HIV testing (*r* = −.11, *p* < .001). Disclosure of sexuality to parents was positively correlated with HIV testing (*r* = .13, *p* < .001) and family bonding (*r* = .20 *p* < .001). Other adult value was positively correlated with family bonding (*r* = .59, *p* < .001).

**Fig. 2:**
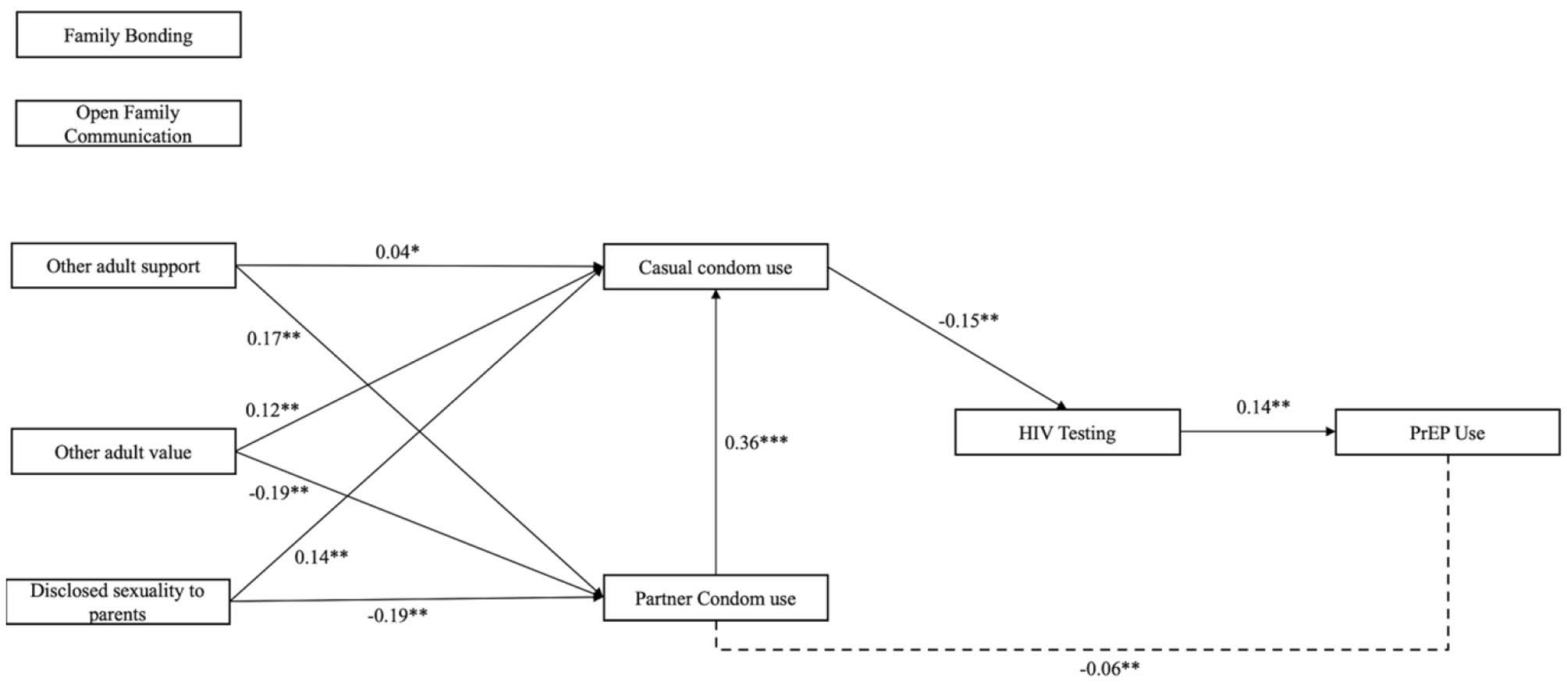
Direct and indirect effects to PrEP use via partner and casual condom use (*N* = 400). **Note**: *p < .05, **p < .01; standardized betas reported, and no lines means non-significance, and broken line mean indirect effects

The path model representing associations between family bonding, open family communication, partner condom use, casual partner condom use, other adult support, other adult value, disclosed sexuality to parents, and HIV testing on PrEP use is presented in Table 4; Fig. 2. The model fit was assessed using the chi-square/df ratio = 43.10, *p* = .061, AIC = 2953.772, and BIC = 3094.150. Partner condom use was positive and directly associated with casual partner condom use (β = 0.36, *p* < .001). Black men’s disclosure of their sexuality to their parents was directly associated with casual partner condom use (β = 0.12, *p* < .001). Having other adults who valued them was directly and positively associated with casual partner condom use (β = 0.14, *p* < .001). Surprisingly, disclosure of sexuality to parents (β = − 0.13, *p* < .05) and having adults who valued them (β = − 0.19, *p* < .01) were both directly and negatively associated with partner condom use. YBMSM who had adults who supported them were directly associated with Black males’ partner condom use (β = 0.17, *p* < .001). Casual partner condom use was both directly and negatively associated with HIV testing (β = − 0.15, *p* < .01). HIV testing was associated with PrEP use (β = 0.14, *p* < .01). Partner condom use was indirectly and negatively associated with PrEP use (β = − 0.06, *p* < .01) (Table 5).

**Table 4:** Direct effects on Preexposure Prophylaxis (PrEP) use (*N* = 400)

	β	B	SE	*P* > z	95% CI
**Casual partner condom use**					
Partner condom use	0.36***	0.22	0.03	0	0.16, 0.29
Family bonding	−0.12	−0.04	0.03	0.25	−0.11, 0.03
Disclosed sexuality to parents	0.12**	0.06	0.03	0.04	0.00, 0.12
Other adult value	0.14*	0.05	0.03	0.04	0.00, 0.10
Other adult support	0.04*	−0.02	0.02	0.04	−0.06, 0.03
Open family communication	−0.00	−0.00	0.03	0.961	−0.06, 0.06
**Partner condom use**					
Family bonding	0.12	0.06	0.06	0.25	−0.05, 0.18
Disclosed sexuality to parents	−0.13**	−0.11	0.05	0.03	−0.20, − 0.01
Other adult value	−0.19**	−0.10	0.04	0.02	−0.19, − 0.02
Other adult support	0.17**	0.09	0.04	0.03	0.01, 0.16
Open family communication	0.03	0.01	0.05	0.772	−0.08, 0.11
**HIV testing**					
Casual partner condom use	−0.15**	−0.21	0.09	0.02	−0.38, − 0.04
Partner condom use	0.06	0.05	0.05	0.30	−0.05, 0.16
**PrEP use**					
HIV testing	0.14**	0.13	0.06	0.02	0.02, 0.25

Note: *p < .05, **p < .01, ***p < .001; β = standardized betas; B = unstandardized betas; *SE* = standard error; CI = confidence interval

**Table 5:** Indirect effects on Preexposure Prophylaxis (PrEP) use (*N* = 400)

	B	SE	*P* > z	95% CI
**HIV testing**				
Partner condom use	−0.01	0.01	0.22	−0.12, − 0.02
Family bonding	0.02	0.01	0.14	−0.01, 0.04
Disclosed sexuality to parents	−0.02	0.02	0.46	−0.06, 0.03
Other adult value	−0.01	0.01	0.16	−0.03, 0.01
Other adult support	0.00	0.02	0.75	−0.06,0.03
Open family communication	0.01	0.01	0.60	−0.02, 0.01
**PrEP use**				
Casual partner condom use	−0.03	0.02	0.46	−0.01, 0.02
Partner condom use	-0.06**	0.03	0.01	−0.01, − 0.12
Family bonding	0.00	0.00	0.32	−0.00, 0.00
Other adult value	−0.00	0.00	0.33	−0.00, 0.00
Other adult support	0.00	0.00	0.75	−0.00, 0.00
Disclosed sexuality to parents	−0.00	0.00	0.52	−0.01, 0.00
Open family communication	−0.00	0.00	0.62	−0.00, 0.00

Note: *p < .05, **p < .01, ***p < .001; B = unstandardized betas; *SE* = standard error; CI = confidence interval

## Discussion

The overarching purpose of the present research was to understand the role of families (both families of origin and chosen families) in facilitating HIV prevention behaviors, such as condom use, HIV testing, and PrEP uptake, among YBMSM aged 18–29 years, using the ecodevelopmental theory as a guide. The ecodevelopmental theory allowed us to situate our findings in the family context of BMSM and how this context impacts their prevention behaviors. Key findings include (1) disclosure of sexuality to parents was positively correlated with HIV testing and family bonding; (2) other adult value was positively correlated with family bonding; and (3) open family communication was positively associated with PrEP use. In keeping with previous research, these findings suggest that the importance of family in promoting HIV prevention behaviors [35] among YBMSM cannot be overstated [36–37].

Additionally, we found support for some of our research hypotheses. First, we hypothesized that family factors (e.g., family bonding and open family communication) would be associated with both casual partner condom use and primary partner condom use. We found that other adult value and open family communication were positively correlated with PrEP use [17, 21]. However, contrary to our expectations, we found that having other adults who value them was directly and negatively associated with partner condom use among YBMSM. It is possible that chosen family members may not necessarily engage in condom use themselves, and therefore, not using condoms may be normalized among these peers. Further research is required to explore assets-based social norms around chosen family and partner condom negotiation among YBMSM [38–39].

Our second hypothesis was that both casual and primary partner condom use would be associated with HIV testing. We found that casual partner condom use was both directly and negatively associated with HIV testing, which suggests that individuals may feel less motivated in seeking regular HIV testing when they are consistently using condoms with casual partners. Nonetheless, these are not mutually exclusive HIV prevention behaviors, and targeted and culturally relevant interventions are still required to identify ways to reduce the perceived burden of additive HIV prevention behaviors on YBMSM with competing priorities or who may have very real barriers to seeking care [40]. Lastly, our third hypothesis was that casual and partner condom use would be indirectly associated with PrEP use, which was supported, though the association was negative. As mentioned before, more research is required on ways to promote additive HIV prevention behaviors rather than replacing some behaviors with others (e.g., taking PrEP because one does not use condoms or getting tested for HIV because one does not use PrEP).

### Limitations

This study is subject to several limitations. First, data are cross-sectional; therefore, a causal link between the outcomes and the exposures cannot be determined. Second, while the items used in our quantitative survey measure very specific behaviors, they are single indicators rather than well-validated measures. (To date, no measures have been developed to assess sexual health communication measures for adolescents, young adults, or their families.) Other constructs that may be of interest were also not included in the present analyses. These might include internalized homophobia, religiosity, and/or resilience.

Next, while our study was designed to assess the needs and experiences of YBMSM, our findings cannot be generalized to all YBMSM or other races of MSM around HIV prevention behaviors. Similarly, the present analyses are limited to MSM based on responses to sex at birth and questions that asked the sex of all sexual partners; thus, participants were not explicitly asked about their sexual orientation. Therefore, the findings do not represent the experiences of nonbinary MSM living with HIV, or the views or experiences of MSM who self-identify as gay, bisexual, or transgender.

### Future implications

Research has consistently shown that supportive relationships, particularly those within the family and with other trusted adults, can have a positive impact on young individuals’ behaviors and choices related to sexual health [17, 21]. Encouraging open and non-judgmental communication about sex, sexual orientation, and HIV prevention within families and other support networks is vital. Providing resources and guidance on how to initiate and maintain such conversations can help bridge the gap and empower young Black gay men to seek information and support from trusted sources. Families and other adult support networks should work towards creating inclusive and affirming environments for young Black gay men. This includes challenging stigma, promoting acceptance, and understanding the unique experiences and needs of this population. Lastly, collaboration between families, community organizations, healthcare providers, and schools is crucial to providing comprehensive support to young Black gay men. These partnerships can help ensure that the necessary resources, services, and education are accessible and tailored to the specific needs of this population.

## Conclusion

Despite these limitations, our study is one of the few that focuses on a rigorous assessment of the role of family structures in promoting HIV prevention behaviors among YBMSM. This conceptualization requires further research, as it promotes assets-based approaches that are conducive to future interventions that promote open communication between families and can help normalize HIV prevention discussion for YBMSM, which has huge implications for their health care-seeking behaviors as they age. Future clinic-based research should include the development of validated measures that assess the adolescent family environment as well as ways in which it impacts health care engagement, care satisfaction, and HIV prevention outcomes. Culturally appropriate approaches to YBMSM are crucial, given that late adolescence and early adulthood are critical periods for HIV incidence and are the developmental stages in which to establish HIV prevention care-seeking behaviors that will carry forward as these individuals age. Overall, recognizing and valuing the role of family and other adult support in HIV prevention among young Black gay men is essential to create environments that support their overall well-being, self-acceptance, and enable access to effective prevention strategies. By working together, we can reduce HIV-related health disparities and promote healthier futures for young Black gay men.

## Data Availability

Data that support the findings of this study are available on request under a license agreement. Written applications can be made to the corresponding author (boyd.465@osu.edu).

## Abbreviations

CI: confidence intervals
HIV: Human immunodeficiency virus infection
US: United States
YBMSM: young Black men who have sex with men
PrEP: Preexposure prophylaxis
